# Upgrading and replacement in CRT devices: experience counts

**DOI:** 10.1007/s12471-015-0772-8

**Published:** 2015-12-07

**Authors:** W.G. de Voogt

**Affiliations:** Pol 20, 2063 JM Spaarndam, The Netherlands

Cardiac resynchronisation therapy has proven to be highly effective in patients with heart failure and an asynchronous contraction pattern of the left ventricle, due to late activation of the posterolateral wall with left bundle branch block configuration on the ECG of more than 150 ms [1].

In pacemaker patients with a high percentage of right ventricular pacing, pacing-induced left ventricular (LV) dysfunction can appear. This newly developing heart failure is most probably the result of pacing-induced LV asynchrony. These patients with pacing-induced heart failure and ICD patients with heart failure who develop a wide QRS complex during follow-up account for the majority of patients who are eligible for an upgrade to a CRT device. In an European survey, up to 25 % of CRT implants were upgrading procedures [2]. The REPLACE registry demonstrated a high percentage of complications in upgrade and revision procedures in 434 CRT patients from 71 centres. It was not stated how many centres actually contributed to this total number of CRT upgrades [3]. Their conclusion ‘pacemaker and implantable cardioverter-defibrillator generator replacements are associated with a notable complication risk, particularly those with lead additions’ made cardiologists somewhat cautious and even reluctant to perform a therapeutic upgrade to CRT. These REPLACE data support careful decision making before device replacement when managing device advisories and when considering upgrades to more complex systems, concluded the authors [3]. And indeed, an upgrade procedure can be complicated and is in need of an experienced skilled operator or at least her or his direct supervision during the procedure. As experience is a major factor in reducing complications during and after device implantation [4], it is reasonable that experience is an even stronger denominator in complicated procedures as primary CRT implants and CRT upgrades.

In this issue, the retrospective data of upgrading procedures to CRT devices were compared with the complications in primary CRT implants during the same period [5]. These retrospective data originate from one tertiary centre with a high patient load. Only three experienced operators performed the majority of procedures or supervised them.

A: Total number of individual implants of 165 (62 %), including 82 upgrade procedures. Thirty assists of which 16 assists in an upgrade procedure.

B: Total number of individual implants of 30 (11 %), including 13 upgrade procedures. Six assists of which 4 assists in an upgrade procedure.

C: Total number of individual implants of 18 (7 %), including 9 upgrade procedures. Three assists of which 0 assists in an upgrade procedure.

Though these data are retrospective, the complication rate is considerably lower when compared with the literature [3]. The most determining factor in the difference of complication rate is most likely skill, which comes from experience and cooperation during these complicated procedures. It was not investigated whether the determining factor was cooperation or experience or its combination.

## How can we perform better than we did before?

In this issue Ter Horst et al. showed that not only experience is a key factor but also inter-collegial consultation during a complicated procedure. Experience is knowledge, derived from expected and particularly unexpected minor and major complications. All the steps in the process of knowledge build-up should not only be written down in the mind of the operator and spread during assist to the next generation of implanting cardiologists in their training period. Though this teaching technique is important and cannot be neglected, we could make a plea for revealing the complications and near-complications that occur. This could add up to a manual showing not only how to perform a certain procedure, but most importantly: how to prevent and cope with complications. This ‘How to avoid complications’ can be written by the pioneers in the field and we can learn from their miss and near-miss experiences and thus enhance the learning curve with less collateral damage and try to achieve the same favourable rate as described by Ter Horst et al. [5].

### Funding

None.

### Conflict of interests

None declared.
